# Short-term clinical efficacy of percutaneous transforaminal endoscopic discectomy in treating young patients with lumbar disc herniation

**DOI:** 10.1186/s13018-018-0759-4

**Published:** 2018-03-20

**Authors:** You-Long Zhou, Gang Chen, Dao-Chi Bi, Xing Chen

**Affiliations:** 1 Department of Orthopedics, Changxing People’s Hospital, Huzhou, 310009 China; 2 Department of Orthopedics, Changxing Branch of 2nd Affiliated Hospital, School of Medicine, Zhejiang University, Hangzhou, 310009 China; 3 Department of Orthopedics, 2nd Affiliated Hospital, School of Medicine, Zhejiang University, 88 Jie fang Road, Hangzhou, 310009 China

**Keywords:** Lumbar disc herniation, Percutaneous transforaminal endoscopic discectomy, Preliminary outcome

## Abstract

**Background:**

In the last decades, full-endoscopic techniques to treat lumbar disc herniation (LDH) have gained popularity in clinical practice. However, few studies have described the safety and efficacy of percutaneous transforaminal endoscopic discectomy (PTED) in treating younger patients with LDH. This study aims to evaluate the preliminary surgical outcome and complication of PTED in treating younger patients with LDH.

**Methods:**

Between June 2012 and June 2016, 72 young patients (< 45 years old) who underwent PTED for single-level LDH were prospectively followed up. All patients were followed up for at least 12 months (range 12–35 months). Pain was measured using visual analogue scale (VAS) scores. Patient satisfaction was evaluated using the MacNab outcome scale. Clinical outcomes were measured preoperatively, at 2 days and 6 months, and 12 months postoperatively.

**Results:**

The mean VAS score for back pain was 5.1 ± 2.3 preoperatively and 3.1 ± 1.2, 2.1 ± 0.5, and 2.0 ± 0.7 at 2 days, 6 months, and 12 months postoperatively, respectively. The VAS score for leg pain was 7.1 ± 2.6 preoperatively and 3.0 ± 1.1, 2.1 ± 1.3, and 1.9 ± 0.8 at 2 days, 6 months, and 12 months postoperatively, respectively. These postoperative scores were all significantly different when compared with preoperative scores (*P* < 0.001). According to the modified MacNab outcome scale, excellent was obtained in 43 patients, good was obtained in 25 patients, and fair was obtained in 4 patients, and 94.44% of these patients had excellent and good outcomes at the final follow-up. There were no complications related to surgery, and no spinal instability was detected.

**Conclusion:**

PTED appears to be an effective and safe intervention for younger patients with LDH. High-quality randomized controlled trials are required to further study the efficacy and safety of PTED in treating younger patients with LDH.

## Background

Lumbar disc herniation (LDH) is a frequently observed orthopedic disease that produces medical and economic burdens to families and society [1, 2]. Most patients with LDH can be cured by conservative treatment, but a considerable number (a reported prevalence of 1–3%) of the patients will eventually undergo surgical treatment [3, 4].

At present, open microdiscectomy remains as the gold standard for treating LDH [5, 6]. In the past decades, significant improvements in the design and use of invasive endoscopic instruments have led to the utilization of full-endoscopic surgical procedures for the treatment of LDH [7]. These endoscopic procedures are expected to be minimally invasive, reduce hospitalization, and shorten recovery time. At present, percutaneous transforaminal endoscopic discectomy (PTED) has become an increasingly popular surgical procedure for treating LDH, since its first application in 1973 [7].

Since its establishment, PTED has been shown to be a promising minimally invasive treatment approach for LDH. The preliminary results of several studies have shown that PTED is effective in patients suitable to undergo this approach, and its clinical outcome is equivalent to traditional open surgery with the added benefit of reduced invasiveness [8–10]. Nevertheless, to the best of our knowledge, few studies have been describing the safety and efficacy of PTED in treating younger patients with LDH. From June 2012 to June 2016, a total of 72 young patients with LDH were treated by PTED in our institution and were continuously followed up for at least 12 months. The present study aimed to evaluate the preliminary surgical outcome and complication of PTED in the treatment of younger patients with LDH.

## Methods

### Clinical data

A total of 72 patients were enrolled into this study. Among these patients, 48 patients were male and 24 patients were female. The age of these patients ranged within 24–45 years old, with an average of 26.3 ± 6.4 years old. The lesions were located at L3/4 in 6 patients, at L4/5 in 31 patients, at L5/S1 in 37 patients, and at both L4/5 and L5/S1 in 2 patients. All 72 patients had lower limb radiating pain and/or low back pain, 18 patients had lower limb intermittent claudication, 42 patients had innervation hypesthesia, and 30 patients had a decrease in muscle strength in corresponding nerves. Among these patients, 60 patients received 3–6 weeks of conservative treatment and had poor or no curative effect before PTED, while the remaining 12 patients with severe acute disc herniation received 2 days of conservative treatment and had no curative effect. All patients were examined by computed tomography (CT) and magnetic resonance imaging (MRI) before PTED, and the diagnosis was confirmed to be combined with the clinical manifestations. Lumbar hyperextension and hyperflexion position X-ray films revealed that there was no lumbar instability in the lumbar vertebrae that were scheduled to operate.

### Surgical methods

#### Surgical instruments

The transforaminal endoscopic system provided by SPINENDOS (Germany) was used in 18 patients, and the transforaminal endoscopic system provided by Shandong Guanlong Medical Supplies Co. Ltd. was used in 54 patients. Furthermore, the radio-frequency electrode system provided by Ellman (USA) was used in 18 patients, and the radio-frequency electrode system provided by Shandong Guanlong Medical Supplies Co. Ltd. was used in 54 patients.

#### Surgical procedures

**Fig. 1:**
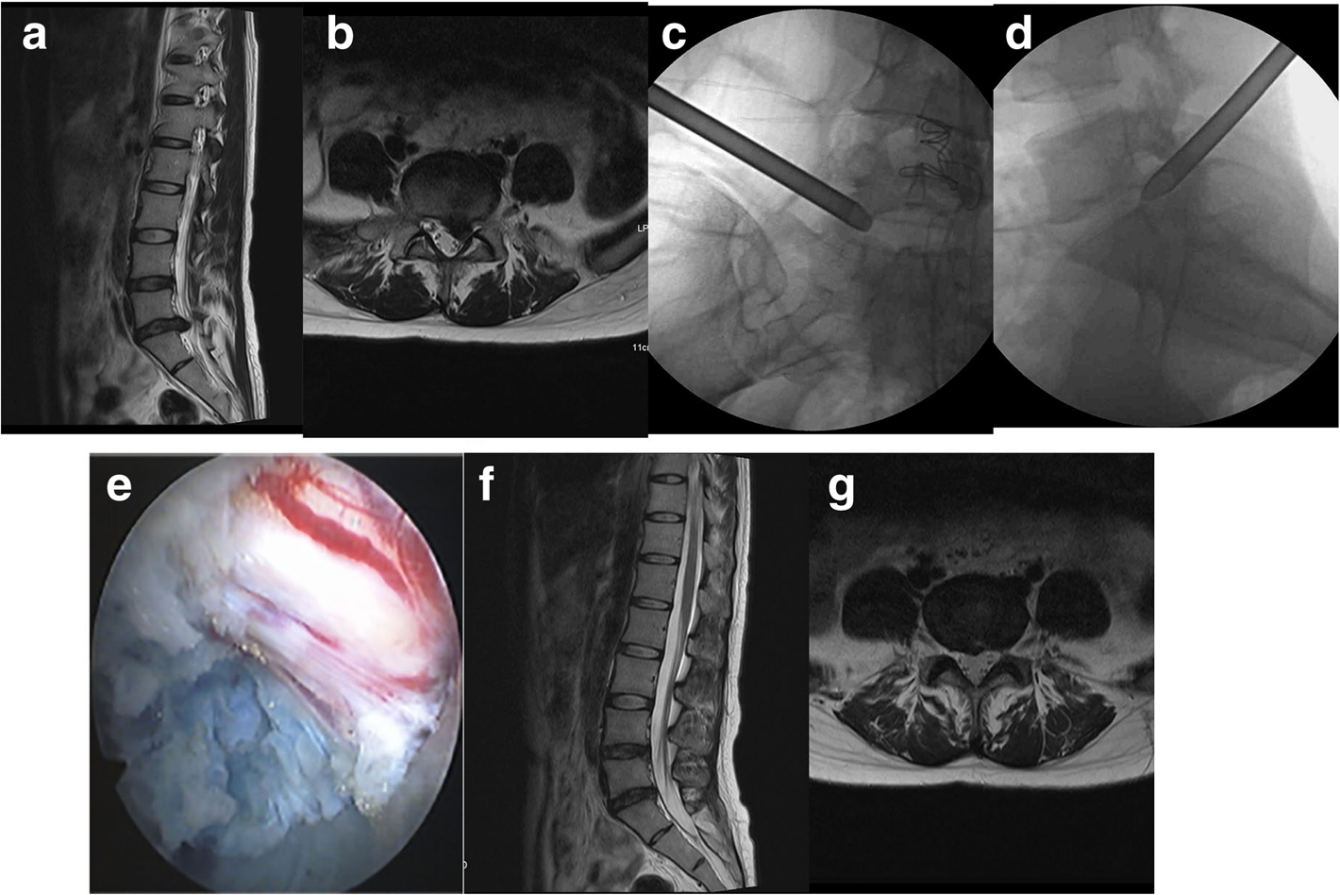
The patient was a 62-year-old male and had a protrusion in the posterior left portion of the L5/S1 interverbral discs. **a** MRI revealed a left L5/S1 interverbral disc protrusion in the sagittal section. **b** In the cross section, it revealed a protrusion in the posterior left portion of the L5/S1 interverbral discs, which compressed the left 1 sacral nerve root. **c** Placement of the working channel during the operation (anteroposterior film). **d** Placement of the working channel during the operation (lateral film). **e** The left five spinal nerve root was exposed during the operation. **f** After the operation, MRI revealed no significant protrusion in the L5/S1 interverbral discs. **g** After the operation, in the transverse section, it revealed no significant protrusion in the L5/S1 interverbral discs, and the nerve roots were not compressed

Patient laid in the lateral position. A soft cushion was placed beneath the waist to make it slightly protrude towards the affected side, in order to increase the height of the affected intervertebral foramen. The labeled operating space was scanned by a C-arm X-ray machine. The computer image processing system was used to measure the distance from the puncture point (for L3/4, the puncture point was 8–12 cm from the middle line; for L4/5 and L5/S1, the puncture point was 12–14 cm from the middle line). After the puncture site was marked, the operation was performed under local anesthesia combined with analgesic drugs. Local infiltration anesthesia was induced by 0.5% lidocaine. The puncture needle was inserted through the entry point, and the skin and fascia above the iliac crest were anesthetized. When the needle reached the bony structure, it was confirmed that the needle had reached the ventral margin of the articular facet of the superior articular process. Then, 2–3 ml of 0.5% lidocaine was locally injected. The puncture needle was slightly bent to make the tip and end of the needle bend towards the ventral side. The puncture needle was slightly pushed to the position between the spinous process and medial margin of the vertebral arch on the anteroposterior X-ray film, while the needle was positioned at the upper edge of the inferior vertebral body on the lateral film. A guide wire was inserted, and the puncture needle was removed. Then, a 0.8-cm long incision was made along the puncture site. A small amount of the tip of the facet of the superior articular process was abraded layer by layer with the aid of the expansion tube, guide rod, and trephine, in order to expand the lateral intervertebral foramen and establish surgical access. After inserting the access, a working channel slope was placed close to the intervertebral disc. A C-arm X-ray machine was used to determine whether the puncture needle entered into the intervertebral space along the channel. Discography was performed using the mixed solution of methylene blue and iohexol at a ratio of 1:9. The presence of exudation of the contrast agent to the spinal canal was observed on the anteroposterior film. The operation for the extirpation of the protruded intervertebral disc, decompression of the nerve root, intradiscal electrothermal annuloplasty, and hemostasis were performed using an endoscope. The degree of nerve root relaxation was determined by the nerve probe and the influence of water pressure on nerve fluctuation. After decompression, the surgical access was pulled out and the wound was sutured (Fig. 1).

#### Postoperative care

Patients had bed rest for 6 h after the surgery and got out of bed to perform appropriate activities with the aid of a waistline. The patients should avoid weight-bearing activities and extreme lumbar flexion, extension, lateral bending, and rotation.

#### Efficacy evaluation

The remission of lumbocrural pain before surgery and at 2 days, 6 months, and 12 months postoperatively was evaluated using the visual analogue scale (VAS), and the recovery of lumbar function 1 year after the surgery was evaluated using the modified MacNab score.

#### Statistical analysis

Data were statistically analyzed using statistical software SPSS 13.0. All results were expressed as mean ± standard deviation ($\overline{x}\pm \mathrm{SD}$). The obtained data were processed by statistical analysis and evaluated using *t* test. *P* < 0.05 was considered statistically significant, and *P* < 0.01 was considered obviously statistically significant.

## Results

All 72 patients were successfully operated. After surgery, one patient had decreased muscle strength in the area innervated by the descending nerve root and hyperalgesia. After conservative treatment, the feeling returned to normal at 2 weeks after the surgery, and muscle strength recovered at 4 weeks after the surgery.

Operation duration was 48–165 min, with an average of 97.5 ± 23.5 min. Six hours after the surgery, all patients were able to get out of bed and perform activities with the aid of a waistline. Hospital stay was 2–7 days, with an average of 3.36 ± 1.52 days. All 72 patients were followed up for 12–35 months. The VAS scores for lumbar pain were 5.1 ± 2.3, 3.1 ± 1.2, 2.1 ± 0.5, and 2.0 ± 0.5 before surgery and at 2 days, 6 months, and 12 months postoperatively, respectively. The VAS scores for lower limb pain were 7.1 ± 2.6, 3.0 ± 1.1, 2.1 ± 1.3, and 1.9 ± 0.8 before surgery and at 2 days, 6 months, and 12 months postoperatively, respectively. The differences in VAS scores before and after surgery were statistically significant (*P* < 0.01). Furthermore, differences in lumbocrural pain scores between 2 days after surgery and 6 months and 12 months postoperatively were statistically significant (*P* < 0.01), while the difference in scores between 6 months after surgery and 12 months after surgery was not statistically significant (*P* = 0.21, Table 1). According to the modified MacNab scale, the curative effect was excellent in 43 patients, good in 25 patients, and acceptable in 4 patients. Patients who achieved excellent and good curative effects accounted for 94.74%. Two patients recurred within 6 weeks after the operation, developed symptoms the same with those before operation, and recovered after the re-operation of transforaminal endoscopic nucleotomy. Recurrence rate was 2.78%.

**Table 1:** VAS scores of lumbar pain and limb pain in different time among 20 cases

Time	VAS scores ($\overline{x}\pm \mathrm{s}$)	
	Lumbar pain	Limb pain
Before surgery①	5.1 ± 2.3	7.1 ± 2.6
2 days after surgery②	3.1 ± 1.2	3.0 ± 1.1
6 months after surgery③	2.1 ± 0.5	2.1 ± 1.3
12 months after surgery④	2.0 ± 0.7	1.9 ± 0.8

① compared with ②③④, *P* < 0.01; ② compared with ③, *P* < 0.01; ② compared with ④, *P* < 0.01; ③ compared with ④, *P* = 0.21

## Discussion

LDH is a common and frequently occurring disease of the spine and is the most common cause of lumbocrural pain. Traditional concepts consider that LDH is a highly occurring disease in middle-aged and elderly populations. In recent years, due to the lifestyle changes of people, the incidence of LDH in young people has increased. A sedentary life causes long-term excessive stress in the waist. When this is coupled with lack of exercise, chronic injury occurs in the lumbar muscles, pathological changes occur in the intervertebral disc, and the spinal structure changes, eventually leading to the occurrence of LDH.

In the surgical treatment of LDH, open nucleotomy through an open window has been used for a long time. However, this approach may induce spinal instability, leading to long-term bed laying. Arthrodesis of the lumbar vertebra has satisfactory curative effects but leads to loss of some of the motor segments of the spine. Furthermore, since young people perform a lot of spinal activities, it has a risk of accelerating the degeneration of the adjacent segments. Scholars have attempted to relieve the symptoms of lumbocrural pain caused by LDH using smaller wounds. In 1975, Hijikata [11] used percutaneous lumbar discectomy (PLD) to treat LDH. In 1989, Schreiber et al. reported the use of endoscopic techniques in the treatment of PLD, in which a working casing was placed in the “safe working triangle area” at the posterolateral side of the interverbral discs, and the decompression of the intervertebral disc was completed under a modified arthroscope [12]. In 1997, Foley reported for the first time that posterior micro-endoscopic discectomy (MED) could be used to treat LDH [13]. Posterior MED was verified to be a truly minimally invasive, direct decompression procedure [3]. However, injuries to the trunk extensors are inevitable [14, 15]. In 1997, Yeung proposed PTED. After its improvement by Hoogland, PTED has been widely promoted and applied at present and is suitable for the treatment of the vast majority of patients with LDH.

PTED is performed under local anesthesia and operates in the safe triangle area of the intervertebral foramen. Surgeons can maintain effective communication with patients. This surgical procedure has high safety and can effectively avoid nerve root injury. This technique uses the lumbar posterolateral puncture approach, the surgical incision is only 0.8 cm long, and this procedure does not damage the lumbar posterior muscles, as well as the important lumbar bone and joint ligament structures. Therefore, this technique will not cause obvious lumbosacral pain and will have no significant effect on lumbar stability. During the operation, there is no need to separate and retract the nerve root and dural sac. Hence, there is no need to disturb nerve tissues in the vertebral canal, and it does not cause significant bleeding and adhesions in the vertebral canal. Furthermore, it has the characteristics of small surgical trauma and fast recovery after the operation [16–18]. In the present study, all 72 patients were able to get out of bed 6 h after the operation, and the average hospitalization time was 3.36 ± 1.52 days.

The clinical effect of transforaminal endoscopic nucleotomy is similar to that of traditional surgery. It can immediately relieve the symptoms of lumbocrural pain. This surgical procedure is gradually being recognized and acknowledged by people. The nerve root is compressed in LDH, and nerve root activity is limited, causing the contracture of ligaments around the nerve root and inducing compression of the nerve root. Inflammatory stimulation of the protruded intervertebral disc leads to scar tissue hyperplasia. Furthermore, it also causes compression of the nerve root. Percutaneous transforaminal endoscopic nucleotomy removes protruded pulpiform nucleus tissues under direct observation, removes scar hyperplasia tissues, and relaxes the nerve root. During the operation, the patient can be relieved of lower limb radiating pain. During the operation, by adjusting the position of the working channel, it allows the operator to directly observe the intervertebral disc and remove loose pulpiform nucleus tissues [19]. During the operation, it should be examined whether the affected nerve root is completely relaxed under an endoscope. The radio-frequency electrode head or special nerve probe can be used to explore the periphery of the nerve and determine whether nerve root pulsations could be observed, understanding the degree of nerve root relaxation through water pressure changes.

Schube et al. [20] reported that a total of 558 patients with LDH underwent PTED, and all patients were followed up for 2 years. The percentage of patients with excellent and good postoperative nerve root VAS scores was 95.3%, no serious complications occurred after operation, no infections occurred in any of the patients, and the recurrence rate was 3.6%. In the present study, differences in back pain and leg pain VAS scores before and after the operation were statistically significant (*P* < 0.01). Furthermore, differences in back and leg pain VAS scores among 2 days, 6 months, and 12 months postoperatively were statistically significant (*P* < 0.01), while differences in back and leg pain VAS scores between 6 and 12 months postoperatively were also statistically significant (*P* < 0.01). According to the modified MacNab scale, the postoperative excellent and good rate was 94.44%. The above results suggest that the short-term curative effect of this surgical procedure is significant, and its postoperative recovery is rapid.

Hirano et al. [21] reported that recurrence rate after PTED was 2.4–8.5%. Furthermore, they considered that the residual intervertebral disc underwent degeneration. When intervertebral stress increased, it extruded at the weakest point of the fibrous rings and posterior longitudinal ligaments, which is the main mechanism of the postoperative recurrence of LDH. During the operation, protrusive and free intervertebral disc pulpiform nucleus tissues should be completely removed as much as possible. In the late stage of the operation, the working channel should be raised to observe the presence of loose pulpiform nucleus tissues in the disc, which should be thoroughly removed. Furthermore, when the endoscope is inserted into the disc, residual pulpiform nucleus tissues on the surface of the endoscope should be coagulated by radiofrequency, in order to reduce early shedding after the operation. After the operation, except for basic daily life activities, patients should lie in bed for 2–3 weeks, try to avoid sneezing and severe coughing, and prevent intestinal obstruction by drug or dietary management. Through the above management, the recurrence rate can be effectively reduced. In the present study, recurrence occurred in two patients within 6 weeks after the operation, and recovery was achieved after performing another transforaminal endoscopic nucleotomy. One patient developed acute protrusion of the L4/5 intervertebral disc caused by increased abdominal pressure induced by severe cough at 2 weeks after the operation, and one patient developed LDH of the operated segment again at 6 weeks after operation. This patient began to work at 20 days after the operation. This was related to the incomplete removal of pulpiform nucleus, as well as premature bending, stooping, and weight-bearing activities.

Nerve root injury is the most common complication of PTED, and its incidence can reach 2.8–17% [22, 23]. It is mainly related to the wound, the squeezing and retraction in the puncture process, placement of the dilator and working casing, or in the abrading and drilling of facet joint. In the present study, one patient developed descending nerve root injury, which was a symptom of severe nerve root stimulation during the process of arthroplasty. However, the patient recovered well after conservative treatment.

## Conclusions

A related literature confirmed that [24] differences in the curative effect and recurrence rate between PTED and conventional posterior interlaminar approach laminectomy were not statistically significant and PTED had a lower incidence of some of the complications. Moreover, during PTED under local anesthesia, surgeons can communicate with patients well, which ensures the safety of the operation. The effectiveness of direct decompression of the spinal canal under direct observation has been confirmed. In the study, all 72 patients achieved good short-term clinical efficacy. However, its long-term efficacy needs further clinical observation.

## Abbreviations

CT: Computed tomography
LDH: Lumbar disc herniation
MED: Micro-endoscopic discectomy
MRI: Magnetic resonance imaging
PLD: Percutaneous lumbar discectomy
PTED: Percutaneous transforaminal endoscopic discectomy
VAS: Visual analogue scale
